# Progress in Surgery

**Published:** 1896-06-06

**Authors:** 


### Progress in Surgery.

### BRAIN SURGERY.—SEQUELÆ OF MIDDLE EAR DISEASE.

**Thrombosis of Cerebral Sinuses.**—Dr. A. Jansen, of Berlin,[1] in a paper on "Sinus Thrombosis following Middle Ear Suppuration" refers to 83 cases gathered from his own practice and from surgical records. Of these 53 were operated upon, with a mortality of about 50 per cent. The chief symptoms of the condition are pyæmic fever with repeated chills, optic neuritis, meningeal symptoms, such as vertigo, restlessness, nausea, rigidity of neck, and stupor; and evidence of spread of the thrombosis along the jugular vein, mastoid vein, or cavernous sinus.

The evidence that the thrombosis is spreading into the jugular vein is found in the firm thickening, with tenderness, along the line of the vein, the enlarged glands, pain on moving the head, and sometimes on swallowing. When the cavernous sinus is becoming involved the eyelids are swollen and the eyeball protrudes. Swelling and tenderness over the mastoid process indicates thrombosis of the mastoid vein.

The writer draws particular attention to the frequent occurrence of abscesses around the sinus. In thirty-five cases of lateral sinus thrombosis he has met with this condition in thirty-one instances. The symptoms of these abscesses are pain on pressure, a swelling behind the mastoid process, often profuse discharge from the ear, and symptoms of general compression. The main risks of sinus thrombosis are septic meningitis and pulmonary embolus, and the indication is to operate early with a view to arresting the spread of the thrombus, and to prevent it becoming septic and purulent. Early operation greatly improves the prognosis.

The author is strongly in favour of exposing and opening the sinus in these cases, but he advises against ligation of the jugular vein as a precautionary measure. When the diagnosis of jugular thrombosis is certain, then ligature is indicated. In operating he uses the chisel in preference to bone forceps or trephine, opens the sinus as far as the thrombus is softened, and excises its wall, and packs with iodoform gauze.

Mr. Mansell Moullin[2] reports three cases illustrative of certain complications of middle ear disease. In one the infection spread forward, giving rise to acute inflammation of the temporal muscle and outer table of the squamous temporal bone. The clinical symptoms closely simulated those of intracranial suppuration or sinus thrombosis, but quickly disappeared after incision of the inflamed temporal muscle. In another case the patient had a septic thrombosis of the lateral sinus following rapidly on an acute otitis media. The only clinical evidences of the condition were stiffness of the neck, paralysis of the external rectus of the opposite eye, rigors, and irregularly high temperature. The third case (the only fatal one) dated the active symptoms from a blow recently received, although there had been discharge from the ear for several years. No thrombus was discovered at operation, but post-mortem examination revealed one in the lateral sinus. This had been the starting point of various septic emboli, which had lodged in the pleura and pericardium, setting up fatal pleurisy and pericarditis.

**Subperiosteal Mastoid Abscess** is a less serious though more obvious complication. Mr. Jackson Clarke[3] reports a case, and makes some remarks on the treatment based on anatomical considerations. He considers that in children it is sufficient, in uncomplicated cases, to open and clear out the antrum without proceeding to throw the antrum and tympanic cavity into one. A post-mortem examination of a patient who died from another cause, three years after having been thus treated, confirms him in this opinion. The disease was completely cured. In obstinate cases of middle ear suppuration the tubercle bacillus should be sought for, and the possibility of syphilis investigated. Each of these when present modified the prognosis unfavourably.

[1] Sammlung klin. Vorträge, No. 130. [2] Lancet, Nov., 1895. [3] Clin. Jour., Feb. 26, 1896.

